# Integrin α2β1 Expression Regulates Matrix Metalloproteinase-1-Dependent Bronchial Epithelial Repair in Pulmonary Tuberculosis

**DOI:** 10.3389/fimmu.2018.01348

**Published:** 2018-06-22

**Authors:** Sara Brilha, Deborah L. W. Chong, Akif A. Khawaja, Catherine W. M. Ong, Naomi J. Guppy, Joanna C. Porter, Jon S. Friedland

**Affiliations:** ^1^Infectious Diseases and Immunity, Imperial College London, London, United Kingdom; ^2^Centre for Inflammation and Tissue Repair, University College London, London, United Kingdom; ^3^Department of Medicine, Yong Loo Lin School of Medicine, National University of Singapore, Singapore, Singapore; ^4^UCL Advanced Diagnostics, University College London, London, United Kingdom

**Keywords:** tuberculosis, extracellular matrix, respiratory epithelial cell, matrix metalloproteinases, integrins

## Abstract

Pulmonary tuberculosis (TB) is caused by inhalation of *Mycobacterium tuberculosis*, which damages the bronchial epithelial barrier to establish local infection. Matrix metalloproteinase-1 plays a crucial role in the immunopathology of TB, causing breakdown of type I collagen and cavitation, but this collagenase is also potentially involved in bronchial epithelial repair. We hypothesized that the extracellular matrix (ECM) modulates *M. tuberculosis-*driven matrix metalloproteinase-1 expression by human bronchial epithelial cells (HBECs), regulating respiratory epithelial cell migration and repair. Medium from monocytes stimulated with *M. tuberculosis* induced collagenase activity in bronchial epithelial cells, which was reduced by ~87% when cells were cultured on a type I collagen matrix. Matrix metalloproteinase-1 had a focal localization, which is consistent with cell migration, and overall secretion decreased by 32% on type I collagen. There were no associated changes in the specific tissue inhibitors of metalloproteinases. Decreased matrix metalloproteinase-1 secretion was due to ligand-binding to the α2β1 integrin and was dependent on the actin cytoskeleton. In lung biopsies, samples from patients with pulmonary TB, integrin α2β1 is highly expressed on the bronchial epithelium. Areas of lung with disrupted collagen matrix showed an increase in matrix metalloproteinases-1 expression compared with areas where collagen was comparable to control lung. Type I collagen matrix increased respiratory epithelial cell migration in a wound-healing assay, and this too was matrix metalloproteinase-dependent, since it was blocked by the matrix metalloproteinase inhibitor GM6001. In summary, we report a novel mechanism by which α2β1-mediated signals from the ECM modulate matrix metalloproteinase-1 secretion by HBECs, regulating their migration and epithelial repair in TB.

## Introduction

Tuberculosis (TB) is a global health problem, with 8.6 million new cases per year, of which an increasing proportion are caused by multi-drug-resistant organisms (1). TB primarily causes disease in the lungs (2). Pulmonary cavitation, characterized by extracellular matrix (ECM) destruction and lung remodeling results in patient morbidity and mortality (3, 4). Pulmonary ECM is composed of a network of molecules including type I, III, and IV collagen, fibronectin, and laminin (5). Type I collagen is the primary structural fibril of the lung, provides tensile strength, and is highly resistant to enzymatic degradation (6). Besides contributing to the biochemical and biomechanical properties of the lung, the ECM also regulates cellular communication and has important roles in regulation of immune responses (7–9).

Matrix metalloproteinases (MMPs) are key in TB-associated tissue destruction and include collagenases, such as MMP-1, which are able to degrade fibrillar type I collagen (10–13). Our group has demonstrated that in pulmonary TB, MMP-1 is the key in the immunopathology of disease and is expressed within TB granulomas and in adjacent airway epithelial cells (10, 14). MMP gene expression is upregulated by diverse stimuli including cytokines and cell–cell interactions (15). MMP activity is specifically inhibited by a family of four tissue inhibitors of metalloproteinases (TIMPs) (16, 17). In TB, cell–cell interactions involving infected and un-infected phagocytic and stromal cells, including respiratory epithelial cells, drive MMP secretion and amplify ECM destruction (18–20).

Despite playing important roles in tissue destruction, MMPs are increasingly recognized as key elements in tissue repair. Following airway injury, cell migration is one of the first events leading to re-epithelialization (21–23). Cell spreading and migration require attachment to the ECM. MMP-1 and 9 may regulate migration of the cells involved in lung epithelial repair by remodeling the ECM (23, 24). MMP-1 has been shown to promote alveolar epithelial cell migration, wound closure, and protection from apoptosis (25). Following TB, tissue repair is key but substantial tissue remodeling may result in cavitation, fibrosis, airway narrowing, and bronchiectasis (3).

Cell adhesion to the ECM is mediated by integrins, which recruit cytoplasmic signaling complexes, comprised of adaptor proteins and signaling molecules, which link integrins to the actin cytoskeleton (26). Such assembly is dependent on combinations of integrin occupancy and/or clustering resulting in a complex hierarchy of downstream events (27), which regulate many aspects of cell function including MMP expression in response to matrix binding (24, 28, 29). Integrin α2β1 and α3β1 bind to collagens and are constitutively expressed by respiratory epithelial cells (30). Integrin α2β1 has a high affinity to type I collagen, while α3β1 also binds other matrix components such as laminins and fibronectin (31, 32).

According to the pattern of expression and activation state, integrins may play important roles in normal tissue homeostasis and tissue repair, or contribute to the development of inflammatory diseases, such as asthma or pulmonary fibrosis (33). For example, the Th2 cytokines IL-4 and IL-13 have been shown to decrease lung epithelial repair in wound healing assays due to decreased expression of integrin α2β1 at the leading edge of epithelial cells (34), while induction of integrin αvβ6 leads to intraepithelial activation of TGF-β and modulation of mast cell protease expression, which may regulate airway responsiveness in allergic asthma. On the other hand, integrin αvβ6 signaling may have a protective role against emphysema, by decreasing MMP-12 expression (35).

In the present study, we investigated the hypothesis that the local ECM has a key role in modulation of MMP secretion from the respiratory epithelium. In our cellular model of pulmonary TB, we investigated the effect on MMP release of integrin-mediated adhesion of epithelial cells to a type I collagen matrix. We demonstrated that, in TB, bronchial epithelial cell adhesion to a collagen matrix is able to modulate both MMP-1 expression and epithelial repair, signaling through α2β1 and the actin cytoskeleton. In contrast, absence of such cell–matrix interactions leads to upregulation of MMP-1, which is known to drive TB immunopathology.

## Materials and Methods

### Reagents

The broad spectrum MMP inhibitor GM6001 was from Calbiochem (Hertfordshire, UK). FITC-conjugated anti-integrin α2 (clone AK7, Abcam, Cambridge, UK), anti-integrin α3 (clone 17C6, Abcam), and IgG1 isotype control antibodies (BD Biosciences, Oxford, UK) were used in FACS assays. Anti-integrin α2β1 (clone BHA2.1) and anti-integrin α3β1 (clone M-KID2) antibodies were from Millipore (Hertfordshire, UK). Rabbit anti-MMP-1 primary antibody (Millipore) and Cy5-goat anti-rabbit IgG (Abcam) secondary antibody were used in confocal assays. Phalloidin conjugated with Alexa Fluor 594 (Thermo Fisher Scientific, Paisley, UK) was used for F-actin staining and DAPI was used as nuclear counterstain. Other reagents were purchased from Sigma-Aldrich (Dorset, UK) unless otherwise stated.

### Cell Culture

Primary human bronchial epithelial cells (HBECs) were cultured in BEGM (Lonza, Basel, Switzerland) according to supplier’s instructions. All experiments were performed between passages 4 and 5. Subcultures were performed when cells were 70–80% confluent and seeded at a density of 42,500 cells/cm^2^. Cells were stimulated with 1:5 dilution of CoMtb or control medium (CoMCont). Cell viability was performed by trypan blue exclusion.

### Conditioned Media Preparation (CoMtb)

CoMtb was prepared as previously described (36). In brief, monocytes from single-donor leukocyte cones (National Blood Transfusion Service, London, UK) were isolated by gradient centrifugation with Ficoll-Paque (GE Healthcare, Buckinghamshire, UK) and adhesion purification. Monocytes were seeded at a density of 2.5 × 10^5^ cells/cm^2^ in 60 mm petri dishes. Non-adherent cells were removed by washing with HBSS (Thermo Fisher Scientific, Paisley, UK). Fresh RPMI medium (Thermo Fisher Scientific) supplemented with 2 mM glutamine and 10 mg/ml ampicillin was added to cells before infecting with Mtb strain H37Rv at a multiplicity of infection of one and incubation overnight at 37°C and 5% CO_2_. Supernatants were collected, filtered through a 0.2 µm polypropylene filter (Whatman Anotop 25, Buckinghamshire, UK), aliquoted, and stored at −20°C. CoMtb contains both host-derived cytokines and chemokines (e.g., TNF-α, IL-1β, and IL-6) and pathogen-derived antigens and lipids (20). CoMCont was prepared in the same manner but in the absence of Mtb infection.

### Coating of Tissue Culture Plates

Tissue culture plates were coated with 100 µg/ml human Coll-I (VitroCol) and blocked with sterile 1% heat denatured BSA. Wells for analysis in the absence of ECM were left uncoated.

Immobilized anti-integrin antibodies can be used as integrin agonists (37–40). Therefore, to selectively analyze engagement of specific integrins, plates were coated with goat anti-mouse IgG monoclonal antibody overnight at 4°C, washed with PBS, and coated for 2 h at room temperature with either 20 µg/ml of anti-integrin α2β1 (clone BHA2.1) antibody or anti-integrin α3β1 (clone M-KID2) antibody, both from Millipore (Hertfordshire, UK).

### Measurement of MMP and TIMP Concentrations

At 24 h post-stimulation, MMP and TIMP concentrations were analyzed by ELISA (Duoset, R&D Systems, Abdingdon, UK) or by Luminex bead array (Luminex 200, Bio-Rad, Hertfordshire, UK) using the Fluorokine MAP kit (R&D Systems) according to manufacturer’s instructions. Lower limits of sensitivity for the Duoset kits are: 21.2 pg/ml for TIMP-1, 31.2 pg/ml for TIMP-2, and 156 pg/ml for MMP-1. In the Fluorokine Luminex kit, the lower limits are: 1.1 pg/ml for MMP-1, 12.6 pg/ml for MMP-2, 7.3 pg/ml for MMP-3, and 13.7 pg/ml for MMP-9. Samples were within the linear range of detection as indicated by the manufacturer.

### Detection of Collagenase Activity

Matrix metalloproteinase-1 collagenolytic activity was measured using EnzChek DQ Coll-I assay (Thermo Fisher Scientific) performed as indicated in the manufacturer’s instructions. Briefly, a standard curve was generated from known concentrations of collagenase from *Clostridium histolyticum* and standards and cell supernatants were incubated with DQ type I collagen for 24 h. Gain of fluorescence originated from DQ collagen degradation. Mean basal collagenase activity from controls was subtracted from CoMtb-stimulated wells without ECM and type I collagen, respectively, and fold-change in collagenase activity was calculated relative to CoMtb-stimulated wells in the absence of ECM.

### Reverse Transcription and Real-Time PCR

After 6 h incubation, HBEC cells were lysed with TRI-reagent and total RNA extracted using PureLink RNA Mini Kit (Thermo Fisher Scientific). 1 µg RNA was reverse transcribed using QuantiTect Reverse Transcriptase Kit (Qiagen, Manchester, UK) and qPCR reactions were performed in the ABI Prism 7700 (Applied Biosystems, Paisley, UK). MMP-1 cycle thresholds were quantified by comparison to an MMP-1 standard curve generated using known MMP-1 concentrations and standardized to 18S rRNA. MMP-1 primers and probes were custom made and supplied by Sigma-Aldrich (forward primer: 5′-AAGATGAAAGGTGGACCAACAATT-3′; reverse primer: 5′-CCAAGAGAATGGCCGAGTTC-3′; probe: 5′-FAM-CAGAGAGTACAACTTACATCGTGTTGCGGCTC-TAMRA-3′) and 18S rRNA primer and probe mix was supplied by Thermo Fisher Scientific.

### Flow Cytometry

HBEC cells were detached with 5 mM EDTA, washed with PBS, fixed with 4% paraformaldehyde (w/v), and blocked with 1% BSA/5% human serum (v/v) buffer. Cells were incubated for 1 h at room temperature with FITC-conjugated anti-human integrin antibody or mouse IgG1 isotype control antibody. Flow cytometry was performed on a FACSCalibur (BD Biosciences, UK) cytometer. The baseline Forward Scatter, Side Scatter, and FL1H settings were adjusted using an unstained, unstimulated cell sample. Mean fluorescence intensities (MFI) were compared after normalization to the isotype control. Data were analyzed using FlowJo vX.0.6 (Tree star, Ashland, OR, USA).

### Confocal Microscopy

4-well glass slides were pre-coated with Coll-I and HBEC cells seeded and incubated as described. Cells were fixed, blocked, and stained with FITC-conjugated anti-integrin or mouse IgG1 isotype control antibody. MMP-1 was stained with a rabbit anti-human MMP-1 primary antibody (Millipore) and Cy5-goat anti-rabbit IgG (Abcam) as secondary antibody. Staining with secondary antibody alone was used as control. For F-actin staining, cells were permeabilized with 0.5% saponin (v/v) and stained with phalloidin conjugated with Alexa Fluor 594 (Thermo Fisher Scientific). DAPI was used as nuclear counterstain. Slides were scanned using a 63× oil immersion objective and to avoid bleed-through effects, each dye was scanned independently in a Leica TCS SP5 confocal microscope equipped with 405 nm diode laser, 488 nm argon laser, 543 and 633 nm HeNe lasers, and using the Leica Application Suite 2.6.2 software (Milton Keynes, UK). Images were edited using ImageJ software v1.46r (NIH, MD, USA).

### Wound Healing Assay

96-well ImageLock plates (Essen Biosciences Ltd., Hertfordshire, UK) were coated with Coll-I or poly-l-lysine and cells were seeded as described. A standard wound was created in each cell monolayer using a WoundMaker (Essen Bioscience Ltd.) according to manufacturer’s protocol. Cells were stimulated and/or soluble Coll-I was added (sColl-I). Plates were incubated in the IncuCyte Zoom (Essen Biosciences Ltd.). Images were taken at 2 h intervals for a maximum of 24 h.

### Histology and Immunohistochemistry

Lung biopsy specimens were collected as part of routine clinical care of patients who were subsequently diagnosed with TB. Patients gave consent for the residual paraffin embedded tissue blocks not required for diagnostic purposes, to be analyzed in this study. The study was approved by the NHS National Research Ethics Service (13/LO/0900) and University College London Hospitals Joint Research Office. Human control or Mtb-infected lung tissue blocks were cut into 5 μm sections and stained with Hematoxylin and Eosin (H&E) or modified Trichrome using an automated slide-stainer (Sakura, Tissue-Tek DRS 2000). In brief, sections were stained with three dyes (Celestine blue/Ferric ammonium sulfate solution, Orange G/Picric alcohol solution or Ponceau 2R/Acid fuchsin solution) to stained collagen (blue), red blood cells (yellow), cytoplasm (red), and nuclei (dark red). Sections were also stained for integrin α2β1 and MMP-1. Anti-α2 integrin antibody (clone EPR5788, Abcam) was diluted 1/500 and applied for 15 min at room temperature following on-board epitope retrieval using Leica Epitope Retrieval Solution 2 (Leica, AR9640, pH9) for 20 min at 99°C. Anti-MMP-1 antibody (clone 41-1E5, Millipore) was diluted 1/600 and applied for 15 min at room temperature following on-board epitope retrieval using Leica Epitope Retrieval Solution 1 (Leica, AR9961, pH6) for 30 min at 99°C. Control sections were treated in exactly the same way as other sections but isotype-dependent non-specific primary antibody binding was controlled for using species- and isotype-matched control antibodies at, at least, the same concentration as that of the primary antibody being controlled for. Specifically, these were a polyclonal rabbit immunoglobulin at 10 μg/ml as control for anti-α2β1; and a mouse IgG2a at 10 μg/ml to control for anti-MMP-1. All immunostaining was carried out on the Leica Bond III automated platform, using the Leica Bond Polymer Refine detection system (Leica, DS9800), which includes a peroxide block, a serum block, and a two-step rabbit anti-mouse post-primary/anti-rabbit polymer amplification system with a DAB chromogen.

All slides were scanned on a Nanozoomer Digital Slide Scanner and images analyzed using NDP.viewer software (both from Hamamatsu Corportation). Sections were inspected by a “blinded” reviewer and five airways from each subject were randomly selected for visual analysis. Representative images were chosen from those selected.

### Statistical Analysis

Data analysis was performed using GraphPad Prism v5.02 (GraphPad Inc., USA). Data are presented as mean ± SD of three replicate samples and are representative of three independent experiments unless otherwise stated. Statistical analysis was performed using one-way ANOVA and Tukey’s range test. For experiments with two independent variables, analysis was performed using a two-way ANOVA and Bonferroni correction for multiple comparisons. Differences between variables were considered statistically significant for *p*-values < 0.05. *p*-Values are represented as follows: *p < 0.05, **p < 0.01, ***p < 0.001; ****p < 0.0001.

## Results

### Bronchial Epithelial Cells on a Type I Collagen Matrix Downregulate CoMtb-Induced Collagenase Activity

**Figure 1:**
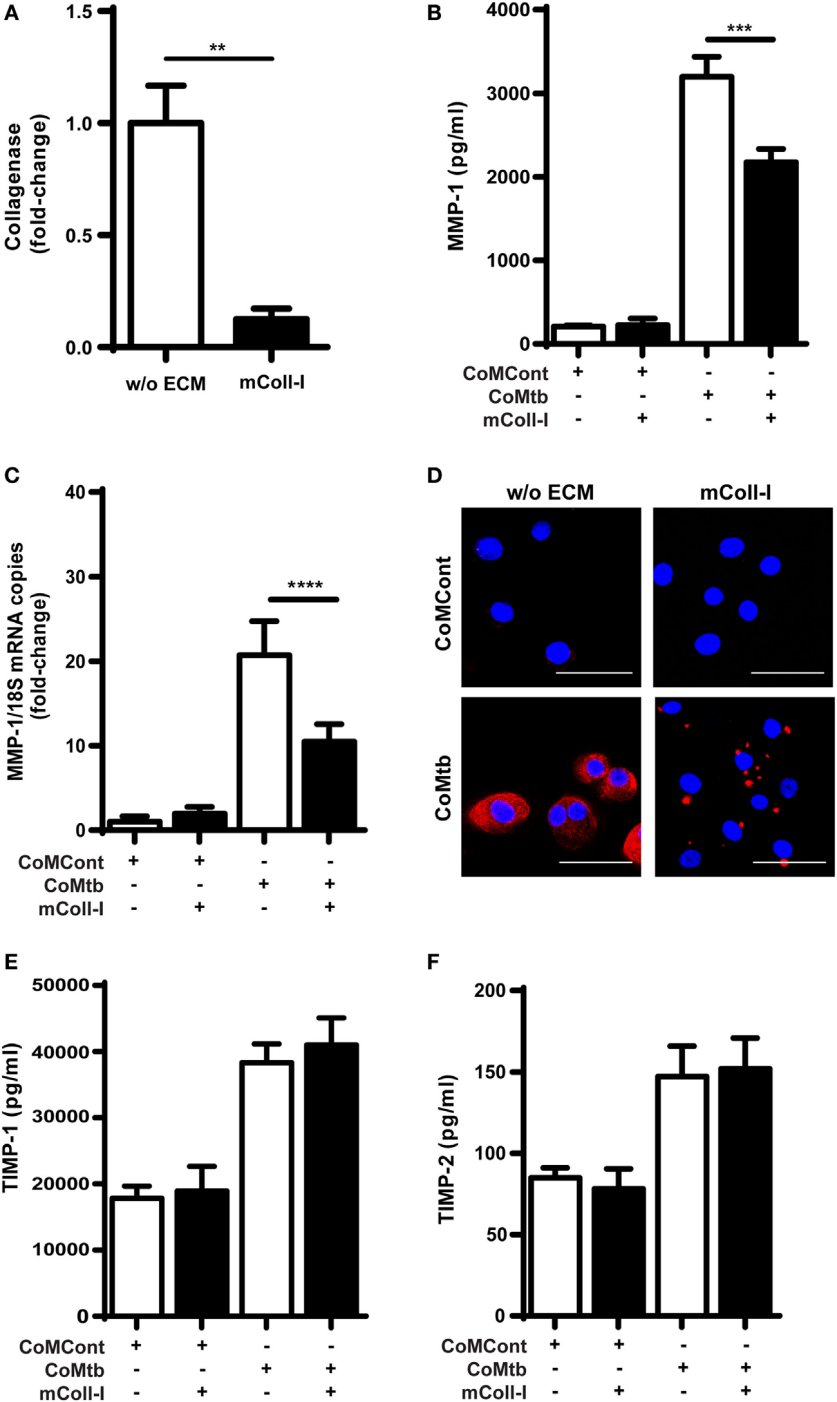
Collagen matrix regulates collagenase activity and matrix metalloproteinase (MMP)-1 expression in CoMtb-stimulated human bronchial epithelial cells (HBECs). HBECs were cultured in the presence of matrix type I collagen (mColl-I) or without extracellular matrix (ECM) and stimulated with CoMtb or control medium (CoMCont). Supernatants were collected at 24 h for analysis of collagenolytic activity and protein secretion, while cell lysates were collected at 6 h for total RNA extraction and analysis of MMP-1 gene expression. **(A)** Fold-change in collagenase activity. Basal collagenase activity in control HBECs was subtracted from respective CoMtb-stimulated wells. Fold-change is relative to mean collagenolytic activity from cells in the absence of ECM. **(B)** MMP-1 secretion (pg/ml). **(C)** Fold-change in MMP-1 mRNA accumulation, normalized to 18S mRNA as reference control. Fold-change is relative to mean MMP-1 expression in control wells without ECM. **(D)** Confocal microscopy images from control (CoMCont) and CoMtb-stimulated HBECs cultured in the presence or absence of matrix Coll-I (*n* = 2). Slides were stained with DAPI (blue) for nucleic acids and anti-MMP-1 primary antibody and Cy5 conjugated anti-rabbit secondary antibody (red). Scale bar: 50 µm. **(E)** Tissue inhibitors of metalloproteinases (TIMP)-1 and **(F)** TIMP-2 secretion. Plots show mean and SD of assays performed in triplicate, which were replicated on three occasions. *p*-Values were obtained with one-way ANOVA with Tukey’s range test. **p < 0.01; ***p < 0.001; ****p < 0.0001.

First, we analyzed respiratory epithelial cell-derived collagenase activity following CoMtb stimulation, both when HBECs were adherent to an intact type I collagen matrix (mColl-I) and in the absence of such matrix. In CoMtb-stimulated HBECs, collagenase activity was decreased by 87.4% when cells were cultured on mColl-I, compared to cells cultured in the absence of ECM (w/o ECM; Figure 1A; *p* < 0.01).

Matrix metalloproteinase-1 is the principal collagenase released by respiratory epithelial cells and associated with tissue destruction in TB (14); therefore, we next investigated this collagenase. CoMtb induced secretion of MMP-1 by HBECs, which was reduced by 32% (*p* < 0.001), when HBECs were cultured on mColl-I (Figure 1B). In addition, mColl-I decreased MMP-1 RNA expression by 49% in CoMtb-stimulated HBECs demonstrating an effect at the gene expression level (*p* < 0.0001; Figure 1C). Furthermore, in HBECs adherent to mColl-I, MMP-1 localization was focal instead of diffuse (Figure 1D).

Matrix remodeling may be critically regulated by MMP:TIMP ratios (15). Therefore, we investigated the effect of adhesion to ECM on secretion of TIMP-1/2, the main secreted inhibitors of MMP-1. Neither the addition of CoMtb nor the presence or absence of mColl-I (Figures 1E,F) affected TIMP-1/2 secretion by HBECs.

### CoMtb-Induced Secretion of MMP-1 by HBECs Is Regulated by Signaling of Integrin α2β1 and the Actin Cytoskeleton

**Figure 2:**
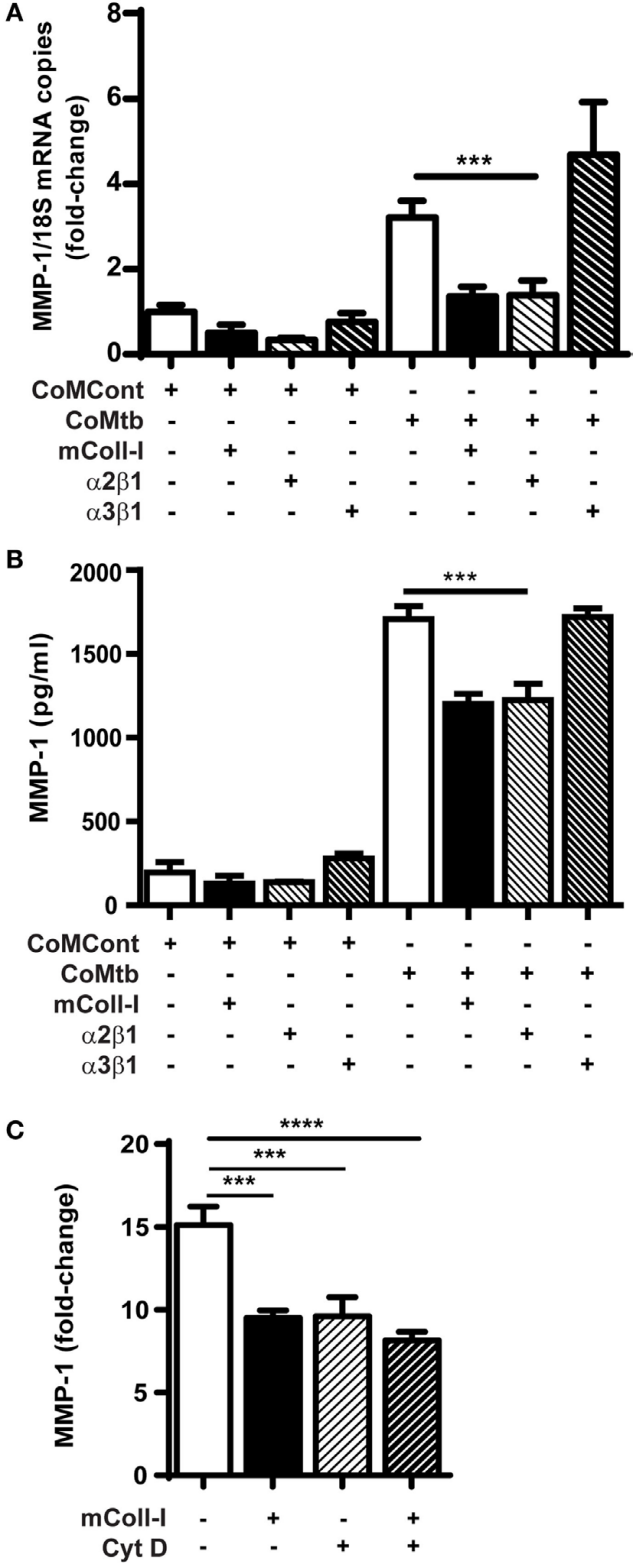
CoMtb-driven matrix metalloproteinase (MMP)-1 gene expression and secretion is regulated by integrin α2β1 and actin cytoskeleton. Human bronchial epithelial cells (HBECs) were seeded in matrix type I collagen (mColl-I) or wells coated with anti-integrin antibodies and stimulated with CoMtb or control medium. **(A)** MMP-1 secretion from HBECs in the presence or absence of coated anti-integrin α2β1 or anti-α3β1 antibodies. **(B)** Fold-change in MMP-1 mRNA accumulation in the presence or absence of coated anti-integrin α2β1 or anti-α3β1 antibodies. MMP-1 mRNA copies were normalized to 18S mRNA and fold-change is relative to mean MMP-1 expression in control wells without extracellular matrix. **(C)** MMP-1 from HBECs pre-incubated for 2 h with 4 µg/ml cytochalasin D before stimulation with CoMtb for 24 h. This figure shows fold-change relative to mean MMP-1 secretion by the respective controls in the absence of CoMtb. Plots show mean and SD of assays performed in triplicate, which were replicated on three occasions. *p*-Values were obtained with one-way ANOVA with Tukey’s range test. ***p < 0.001; ****p < 0.001.

Next, we investigated the possibility that signaling through collagen-binding integrins expressed by HBECs might regulate MMP-1. Plates were either left uncoated, or coated with mColl-I, with antibodies to α2β1 or with antibodies to α3β1 integrins. CoMtb-stimulated HBECs adherent to anti-α2β1 antibodies had decreased MMP-1 gene expression and secretion to cells adherent to mColl-I. In contrast, adhesion to coated anti-α3β1 antibodies resulted in high level MMP-1 gene expression and secretion, similar to that observed in the absence of ECM (Figures 2A,B).

The actin cytoskeleton is an important component of downstream integrin signaling. To examine cytoskeletal involvement in CoMtb-stimulated α2β1-dependent MMP-1 secretion, we next inhibited F-actin polymerization using 4 µg/ml cytochalasin D (Figure 2C). F-actin inhibition decreased the baseline MMP-1 secretion after CoMtb stimulation by approximately 30% (from 3,198.4 ± 238 to 2,229.7 ± 269 pg/ml; *p* < 0.001) in HBECs not adherent to ECM. In addition, Cytochalasin D prevented further inhibition of MMP-1 secretion when cells were cultured on mColl-I, which suggests a role for the actin cytoskeleton in the α2β1-mediated inhibition of MMP-1 expression.

**Figure 3:**
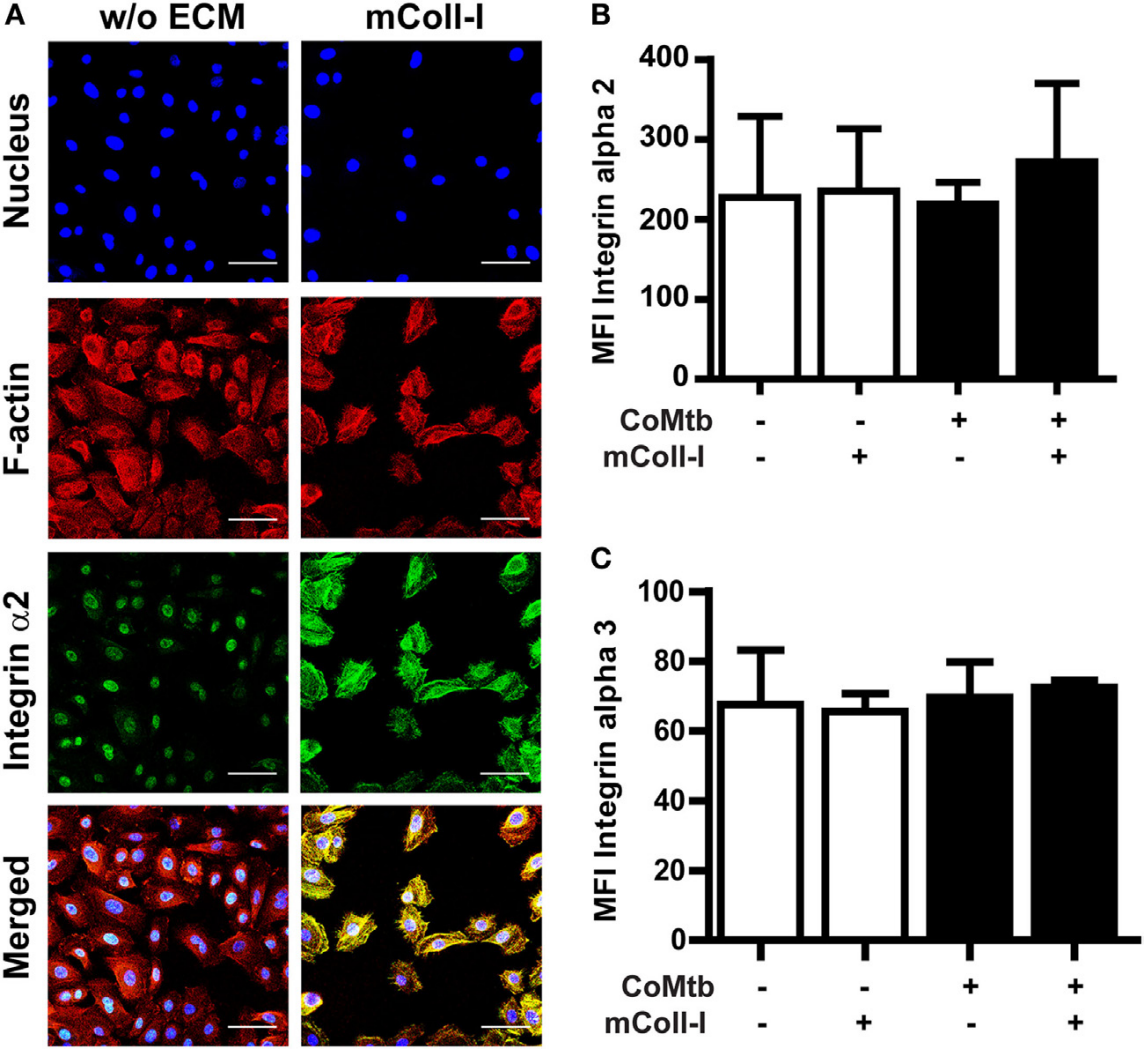
Matrix type I collagen induces α2β1 integrin clustering and actin colocalization without affecting integrin surface expression. Human bronchial epithelial cells (HBECs) were cultured in the in the presence of matrix or soluble Coll-I or without extracellular matrix (ECM) as controls and stimulated with CoMtb or control medium. **(A)** Confocal microscopy figures of CoMtb-stimulated HBECs. Cells were stained with DAPI (blue) for nucleic acids, phalloidin conjugated with Alexa Fluor 594 (red) for F-actin, and FITC-conjugated anti-integrin α2 antibodies (green) for integrin subunit α2. Yellow color shows co-localization of F-actin and integrin α2β1 in merged images. Scale bar: 50 µm. **(B,C)** Control and CoMtb-stimulated HBECs were labeled with FITC-conjugated anti-integrin α2 or α3 and analyzed by FACS. FITC-conjugated mouse IgG1 was used as isotype control. Plots show mean fluorescence intensities (MFI) and SD for **(B)** α2 and **(C)** α3 integrins of three independent experiments.

Next, we investigated the effects of CoMtb on the surface expression and distribution of integrin α2β1, as well as F-actin polymerization with adhesion to collagen. Confocal microscopy revealed that in the absence of ECM, CoMtb-stimulated HBECs had a lower signal from integrin α2β1 (Figure 3A). In HBECs cultured on mColl-I, integrin α2β1 was mainly at the cell–matrix interface co-localizing with F-actin.

However, there were no significant differences in MFIs by FACS for the α2 integrin subunits in the presence or absence of matrix (Figure 3B), indicating that the increase in signal detected by confocal microscopy was principally due to clustering of integrins. As expected, no differences were seen in expression of the α3 subunit (Figure 3C).

These results indicate that the modulation of MMP-1 gene expression and secretion by mColl-I in CoMtb-stimulated HBECs is not due to increased expression of integrin α2β1, but due to matrix-induced clustering of integrin α2β1 and subsequent actin polymerization at the sites of adhesion.

### Airways With Reduced Bronchial Collagen Matrix Appear to Have Further Increased Epithelial MMP-1 Expression in Lung Biopsies of TB Patients

**Figure 4:**
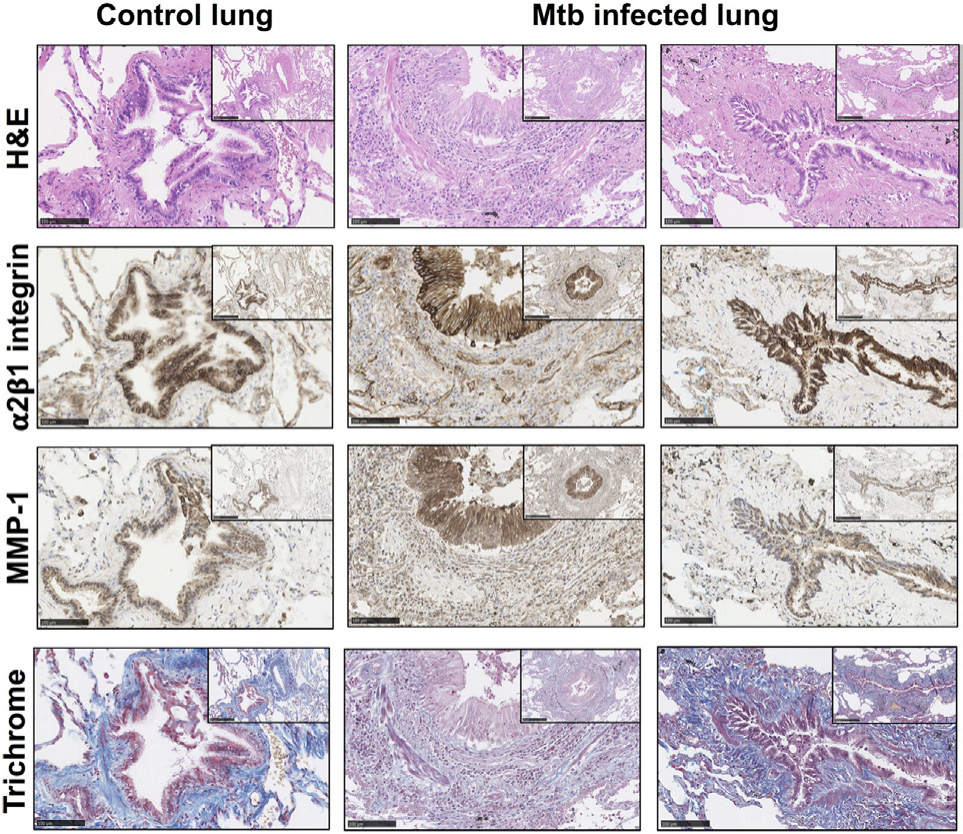
Matrix metalloproteinases (MMP)-1 is further increased in lung tissue sections with altered collagen staining from Mtb-infected patients. Paraffin-embedded lung tissue blocks from Mtb (*n* = 5) and control (*n* = 2) patients were sectioned into 5 µm slices and stained for Hematoxylin and Eosin (H&E), integrin α2β1 (1/500 anti-α2 antibody, clone EPR5788) (brown), MMP-1 (1/600 anti-MMP-1 antibody, clone 41-1E5) (brown), and modified Trichrome stain, which stains collagen (blue). Sections were inspected by a “blinded” reviewer and five airways from each individual were randomly selected for visual analysis. Staining of α2β1 (brown) was similar between Mtb patients and controls but MMP-1 (brown) is increased in Mtb patients. This figure shows representative images from a control lung (left panels) and two Mtb lungs (middle and right panels). Images are shown at 20× with inset shown at 10× magnification. Images were analyzed with the NDP.viewer software. Scale bars: 250 µm for images at 10× and 100 µm for images at 20×.

To investigate the relevance of these findings in human TB infection in patients, type I collagen and integrin α2β1 expression were analyzed in lung biopsies from TB patients (*n* = 5) compared with control lung sections (*n* = 2). Clinical data on the TB patients and controls are summarized on supplementary Table S1 in Supplementary Material. Tissue remodeling was seen in association with and adjacent to bronchial epithelial cells in TB as demonstrated by H&E staining (Figure 4; Figures S1 and S2 in Supplementary Material). The bronchial epithelium stained strongly for integrin α2β1 and, consistent with data from cellular studies, overall staining of integrin α2β1 was similar between controls and TB patients. Furthermore, in biopsies from TB patients, there were airways with altered collagen matrix compared to controls (middle and right hand panels), and areas of increased MMP-1 staining.

### HBEC Interactions With a Type I Collagen Matrix Enhances Cell Migration and Wound Repair in TB

**Figure 5:**
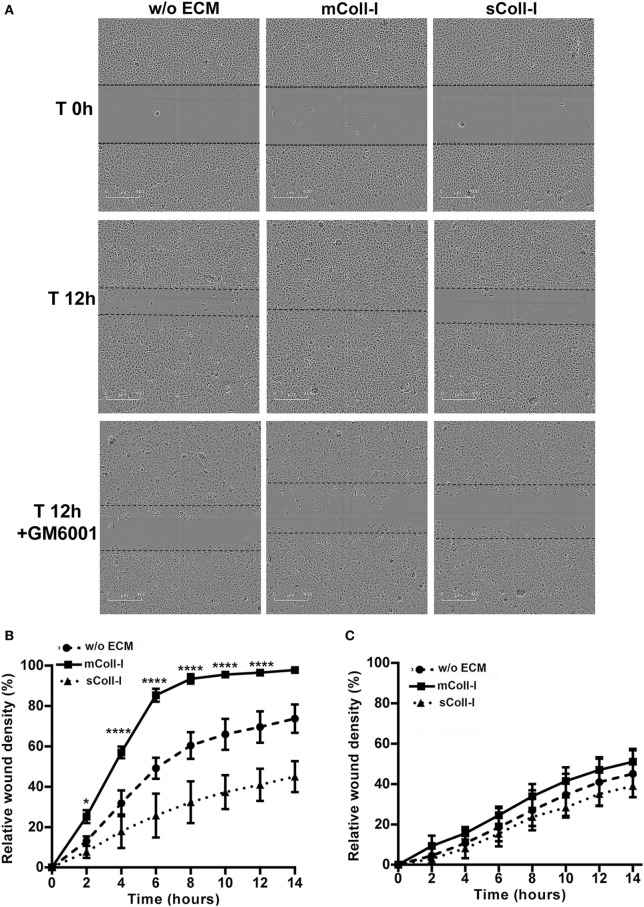
Type I collagen matrix enhances respiratory epithelial cell migration and wound repair in TB. Human bronchial epithelial cells (HBECs) were cultured in the absence of matrix [w/o extracellular matrix (ECM)] or in the presence of matrix type I collagen (mColl-I). Wells without mColl-I were pre-coated with poly-l-lysin. Collagen in solution was also added as control (sColl-I). Cells were stimulated with CoMtb with or without 10 µM GM6001. Images were acquired at 2 h intervals from wound creation and figures shown time 0–14 h. **(A)** Images acquired at time 0 and 12 h post-wound creation from CoMtb stimulated HBECs w/o ECM, with mColl-I or sColl-I and 10 µM GM6001. Scale bars: 400 µm. Relative wound density coverage was analyzed for wells with: **(B)** CoMtb; or **(C)** CoMtb and 10 µM GM6001chemical inhibitor. Relative wound density correspond to the amount of wound coverage by migrating cells. Plots show means and SD of four replicate wells and are representative of at least two independent experiments. *p*-Values were obtained with a two-way ANOVA, with Bonferroni correction for multiple comparisons. *p-value <0.05, ****p-value < 0.0001.

Finally, to investigate the functional consequences of adhesion to an intact matrix in TB, a model of bronchial epithelial wound healing was developed using HBECs either adherent to mColl-I or cultured in the absence of ECM (poly-l-lysine coated wells). Following stimulation by CoMtb, HBECs cultured on mColl-I migrated faster than cells cultured in the absence of ECM, taking approximately 14 h to close the wound (Figures 5A,B). No difference in the rate of migration was seen in controls between the conditions (Figure S3 in Supplementary Material). To confirm that an intact matrix was required for this increase in cell migration, HBECs were also cultured on poly-l-lysine and type I collagen was added in solution (sColl-I). In the presence of sColl-I, HBECs migrated even more slowly than the corresponding cells in the absence of ECM, with only a 45% relative wound coverage after 14 h. Migration was mediated by MMP activity, since pretreatment of cultures with 10 µM GM6001 inhibited cell migration in the presence or absence of mColl or sColl (Figure 5C).

## Discussion

In TB, epithelial repair is a key process in healing from active TB, which may follow extensive destruction of the ECM. Previous studies have identified MMP-1 activity, from multiple cell types, including respiratory epithelial cells, as having a key role in tuberculosis-associated tissue destruction (10, 14). In this study, we demonstrate that the interaction between the cell and the collagen matrix is an important factor regulating respiratory epithelial response to Mtb-infection. In particular, we describe a process by which immobilized mColl-I modulates MMP-1 secretion, activity, and localization in response to CoMtb, *via* integrin α2β1 and the actin cytoskeleton. This is consistent with accumulating evidence that the ECM environment is at least as important in regulation of cellular function as soluble factors (41).

Our data show that respiratory epithelial cell MMP-1 gene expression and secretion are modulated by cell adhesion to the ECM. Adhesion to type I collagen was associated with decreased collagenolytic activity and MMP-1 gene expression, and pericellular, focal localization. The secretion of MMP-1 inhibitors -TIMP-1/2- was not decreased by CoMtb-stimulation of HBECs, which is likely to further contribute to matrix preservation.

The effect on CoMtb-driven MMP-1 expression following airway epithelial adhesion to matrix collagen was integrin dependent. MMP-1 secretion was regulated by integrin α2β1 but not by integrin α3β1, the other collagen-binding integrin expressed on respiratory epithelial cells. The presence of CoMtb did not alter the surface expression of integrin α2β1 and so the effects on MMP-1 expression appear to be due to events after ligand binding. Furthermore, cytochalasin D, which blocks actin polymerization, reduced baseline MMP-1 secretion, but no further reduction was seen when cells are cultured on mColl-I. Thus, MMP-1 secretion seen when HBECs are stimulated with CoMtb appears to be dependent on actin polymerization, and modulated by integrin α2β1 signaling. In migrating keratinocytes, there is direct binding of pro-MMP-1 to integrin α2β1. The ternary complex formed between integrin α2β1, type I collagen, and pro-MMP-1 spatially confines proteolysis to specific points of cell-matrix contacts, which allows directed cell migration and re-epithelization, and this may also be important for bronchial epithelial preservation in TB (42–44).

It is well known that mechanical changes in contractile protein expression allow epithelial cells to respond to the mechanical properties of the ECM during tissue repair. These changes are brought about by integrin signaling from the ECM environment (outside-in). Integrin signals can activate different pathways such as the extracellular signal-regulated kinase (ERK)/mitogen-activated protein kinase (MAPK) or the PI3K/Akt pathways (45), and these pathways are also involved in upregulation of MMP expression in tuberculosis (18, 36). One study investigating modulation of MMP-9 by α3β1 integrin in malignancy, using immortalized mouse keratinocytes, demonstrated that α3β1 signaling leads to an increase in MMP-9 mRNA stability by activation of the MEK/ERK pathway (46).

In a study using airway smooth muscle (ASM) cells from asthma and control patients, stimulation with Tenascin-C induced greater MMP-1 expression by asthma patient ASM-derived cells, *via* JNK, p38, and ERK1/2 MAPKs signaling, and blocking β1 and β3 integrins decreased this response (47). Downstream of integrin α2β1, Cdc42 signaling was shown to decrease MMP-1 expression through Erk-1/2 inhibition in activated keratinocytes and dermal fibroblasts on type I collagen (48, 49). Integrins may also activate the PI3K pathway through the focal adhesion kinase, and our group has previously shown that PI3K is an important negative regulator of MMP-1 and its blockade leads to MMP-1 upregulation in CoMtb-stimulated NHBE cells (36). Together, these studies indicate that changes in the ECM due to chronic inflammation or infection can affect integrin signaling and MMP expression, which ultimately can impact lung repair. In particular, MMPs are upregulated as cells transition into a more motile phenotype but are downregulated by factors that promote cell tension and actin formation, such as a collagen ECM; however, the precise mechanisms by which this is achieved is unclear and the subject of future work.

The cellular data were supported by findings in lung biopsies from TB patients, where staining of integrin α2β1 was clearly demonstrated in TB. Integrin α2β1 expression levels were similar between the bronchial epithelium of controls and TB patients consistent with there being no changes in total α2β1 detected in the cellular system. MMP-1 expression was increased in the bronchial epithelium of TB patients with some reduction in subepithelial collagen ECM staining compared to control subjects. This may be due to inflammatory MMP activity causing tissue destruction and a subsequent reduction in the α2β1 integrin-mediated regulation of MMP-1 that we have demonstrated *in vitro*.

Airway epithelium repair is a complex process, and cell–matrix interactions have to be strictly controlled to confer an adequate cell adhesion to the ECM (50). CoMtb-stimulated HBECs seeded on a type I collagen matrix had a greater migratory ability in a wound healing assay than cells in the absence of ECM. Both integrin occupancy and clustering by immobilized matrix type I collagen were required for this response since addition of type I collagen in solution (sColl-I) decreased HBECs migratory ability when compared with cells adherent to mColl-I. A study investigating the role of MMP-7 and Sydecan-1 in lung repair, using organotypic airway cultures, reported that the activation state of α2β1 integrin can either promote or prevent airway epithelial cell migration and wound closure (51), which indicates that for migration and re-epithelization, the type of interaction between the integrin α2β1 and the ECM may induce different downstream signaling and actin reorganization, which can either cause cell spreading or promote cell migration.

The fact that sColl had quite different effects in this model of TB and did not drive respiratory epithelial repair suggests that destroyed collagen, characteristic of severe human disease, may lead to further tissue damage and not healing. These findings are consistent with those reported in aged skin in which fragmentation of collagen with age leads to increased MMP-1 secretion, and further skin damage (52).

Proteolysis of the ECM is necessary for cell migration, but in TB, excessive proteolysis of the ECM is associated with tissue damage (4, 53). The differential effects of MMP-1 secretion mediated by mColl-I and integrin α2β1 clustering show a previously unrecognized role for the ECM in tissue repair in TB. These findings are consistent with studies in dermal wounds, where MMP-1 secretion may promote cell migration, while high-level expression of MMP-1 impedes tissue repair and are associated with chronic non-healing wounds (49, 54). Here, we show that although type I collagen reduces MMP-1 expression, the MMP-1 is more focally concentrated. Integrin α2β1 is known to bind MMP-1 (42), and this may concentrate the collagenolytic activity at the leading edge of the cell. By binding both the substrate (collagen) and the enzyme (MMP-1), the integrin is able to focus the collagenase activity and promote wound healing while reducing bystander tissue destruction.

In summary, our data show that, in TB, an intact collagen matrix signaling through the α2β1 integrin and the actin cytoskeleton is important in bronchial epithelial cell migration and tissue repair. Understanding such mechanisms involved in the balance of tissue destruction and repair in TB is crucial for development of new therapeutic approaches capable of tackling immunopathology, and to decrease morbidity and mortality when used in combination with anti-tuberculous therapy.

## Ethics Statement

Lung biopsy specimens were collected as part of routine clinical care of patients who were subsequently diagnosed with TB. Patients gave consent for the residual paraffin embedded tissue blocks not required for diagnostic purposes, to be analyzed in this study. The study was approved by the NHS National Research Ethics Service (13/LO/0900) and University College London Hospitals Joint Research Office.

## Author Contributions

SB, JP, and JF conceived the project, designed the research, analyzed the data, and wrote the manuscript. SB, DC, AK, CO, and NG performed all experiments. All authors revised the manuscript and approved its final version.

## Conflict of Interest Statement

The authors declare that the research was conducted in the absence of any commercial or financial relationships that could be construed as a potential conflict of interest.
