# Supplementary material for: Integrin α2β1 Expression Regulates Matrix Metalloproteinase-1-Dependent Bronchial Epithelial Repair in Pulmonary Tuberculosis
Source: Front Immunol. 2018 Jun 22;9:1348. doi: 10.3389/fimmu.2018.01348 (PMC6024194; doi:10.3389/fimmu.2018.01348)
Supplement: Supplementary file 1 [file Image_1.PDF]

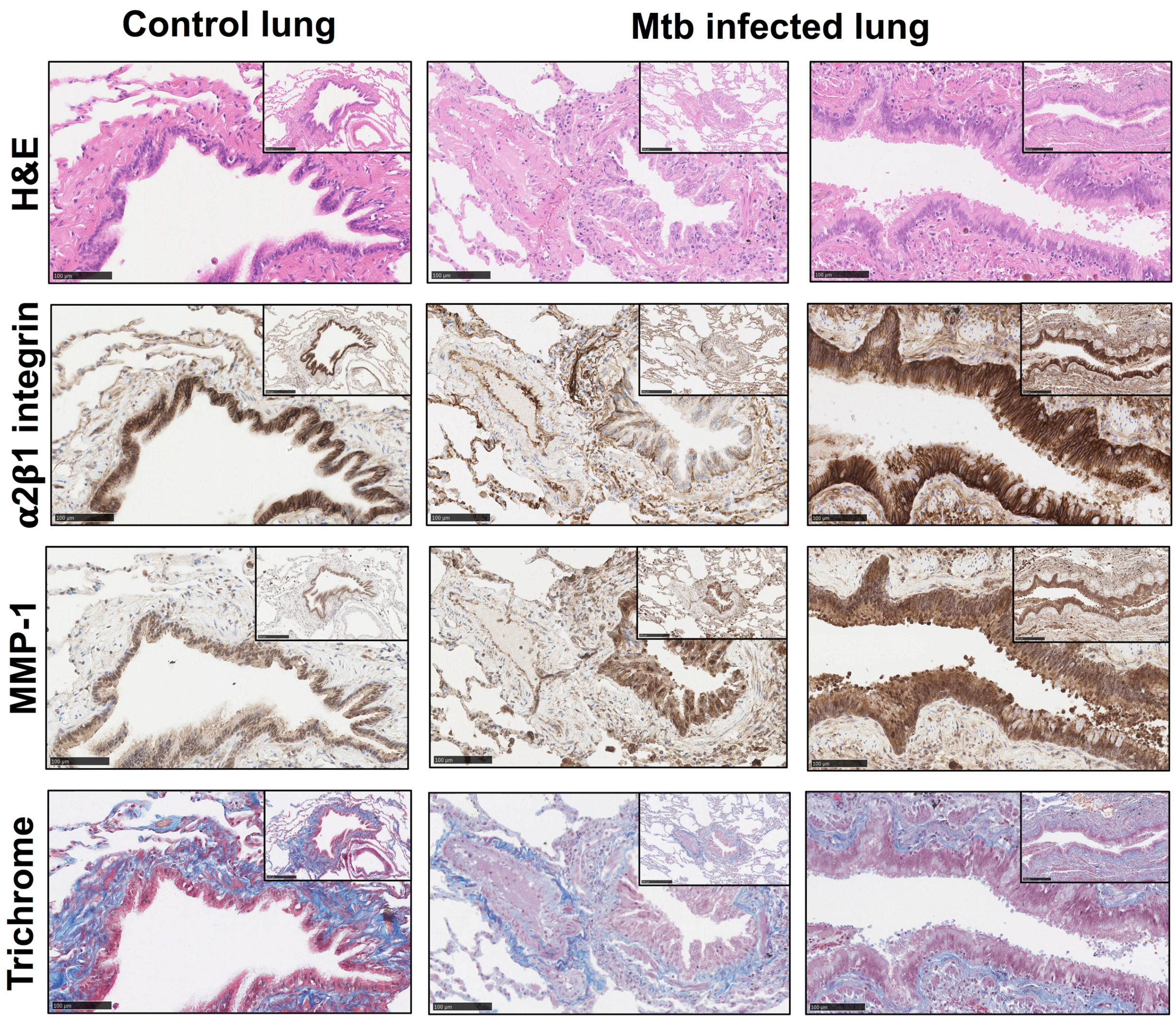

Figure S1- MMP-1 is increased in lung tissue sections with altered collagen staining from Mtb-infected patients.

Paraffin embedded lung tissue blocks from Mtb (n=5) and control (n=2) patients were sectioned into 5µm slices and stained for Hematoxyline and Eosin (H&E), integrin α2β1 (1/500 anti- α2 antibody, clone EPR5788) (brown), MMP-1 (1/600 anti-MMP-1 antibody, clone 41-1E5) (brown), and modified Trichrome stain which stains collagen (blue). Sections were inspected by a 'blinded' reviewer and 5 airways from each individual were randomly selected for visual analysis. Staining of α2β1 (brown) was similar between Mtb patients and controls but MMP-1 (brown) is increased in Mtb patients. Figure shows representative images from a control lung (left panels) and 2 Mtb lungs (middle and right panels). Images are shown at 20x with inset shown at 10x magnification. Images were analyzed with the NDP.viewer software. Scale bars: 250µm for images at 10x and 100µm for images at 20x.
