# Supplementary material for: Integrin α2β1 Expression Regulates Matrix Metalloproteinase-1-Dependent Bronchial Epithelial Repair in Pulmonary Tuberculosis
Source: Front Immunol. 2018 Jun 22;9:1348. doi: 10.3389/fimmu.2018.01348 (PMC6024194; doi:10.3389/fimmu.2018.01348)
Supplement: Supplementary file 2 [file Image_2.PDF]

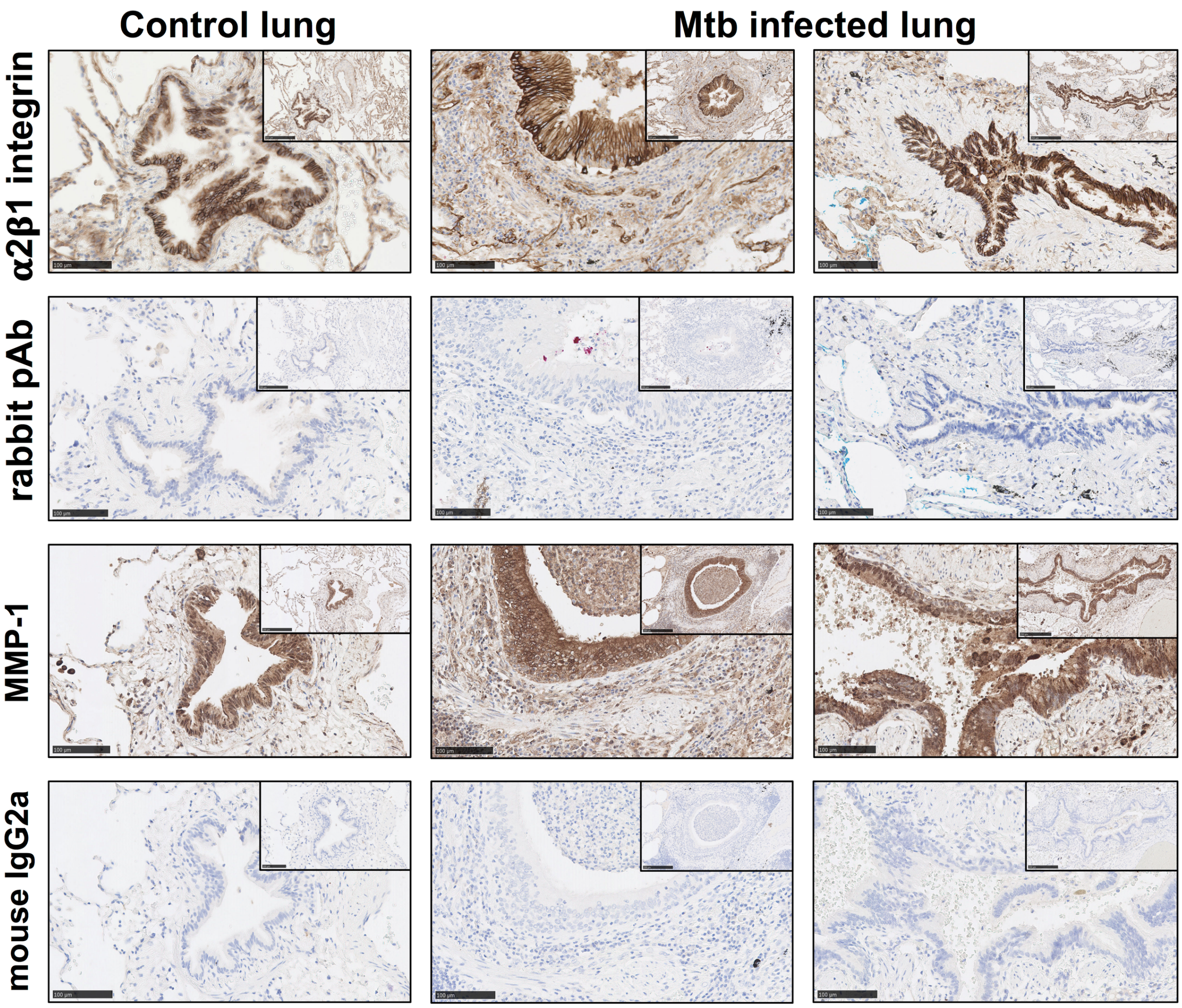

Figure S2- Staining of integrin α2β1, MMP-1 and respective isotype controls from control and Mtb-infected patients.

Paraffin embedded lung tissue blocks were sectioned into 5µm slices and stained for integrin α2β1 (1/500 anti-α2 antibody, clone EPR5788), rabbit isotype control antibody (pAb; 10ug/ml), MMP-1 (1/600 anti-MMP-1 antibody, clone 41-1E5), and mouse isotype control antibody (10ug/ml). Isotype-dependent non-specific primary antibody binding was controlled for using a polyclonal rabbit immunoglobulin as control for anti-α2β1 and a mouse IgG2a to control for anti-MMP-1. Images are shown at 20x with inset shown at 10x magnification and were analyzed with the NDP.viewer software. Scale bars: 250µm for images at 10x and 100µm for images at 20x.
