# Supplementary material for: Integrin α2β1 Expression Regulates Matrix Metalloproteinase-1-Dependent Bronchial Epithelial Repair in Pulmonary Tuberculosis
Source: Front Immunol. 2018 Jun 22;9:1348. doi: 10.3389/fimmu.2018.01348 (PMC6024194; doi:10.3389/fimmu.2018.01348)
Supplement: Supplementary file 3 [file Image_3.PDF]

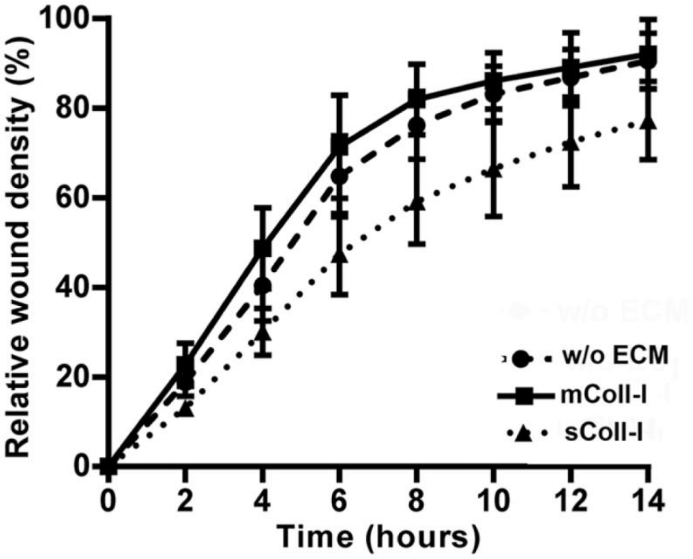

Figure S3- Respiratory epithelial cell migration and wound repair in control HBECs. HBECs were cultured in the absence of matrix (w/o ECM) or in the presence of matrix type I collagen (mColl-I). Wells without mColl-I were pre-coated with poly-L-lysin. Collagen in solution was also added as a control (sColl-I). Cells were stimulated with control medium. Images were acquired at 2 hours intervals from wound creation and figures shown time 0 to14 hours. Relative wound density correspond to the amount of wound coverage by migrating cells. Plots show means and SD of four replicate wells and are representative of three independent experiments. Statistical analysis was performed with a two-way ANOVA, with Bonferroni correction for multiple comparisons.
