# Supplementary material for: Integrin α2β1 Expression Regulates Matrix Metalloproteinase-1-Dependent Bronchial Epithelial Repair in Pulmonary Tuberculosis
Source: Front Immunol. 2018 Jun 22;9:1348. doi: 10.3389/fimmu.2018.01348 (PMC6024194; doi:10.3389/fimmu.2018.01348)
Supplement: Supplementary file 4 [file Table_1.PDF]

Table S1: Clinical details of patients undergoing lung biopsy.

Patient	Operation	Age at time of surgery	Gender	Ethnicity	Risk factors for MTB	Histology
1	Wedge resection of cavitating nodule.	62	Male	South Asian	Diabetic on metformin<br>RA on MTX and prednisolone	Necrotizing granulomas and multinucleate giant cells
2	Wedge resection of one of multiple nodules.	56	Female	Chinese	B cell lymphoma in remission. Father and grandmother had TB	Necrotizing granulomas
3	Wedge resection of nodule	32	Female	Turkish	None	Necrotizing granulomas
4	Wedge resection of apical lung lesions	48	Female	White British	None	Necrotizing granulomas and MNGC
5	Wedge resection of solitary lesion	33	Male	African	None	Necrotizing granulomas with caseation in lung and associated lymph-nodes.
Control 1	Right middle lobectomy for malignant nodule adenocarcinoma	70	Female	Not stated	None	Adenocarcinoma, full resected
Control 2	Wedge resection of solid non-malignant nodule	49	Female	White British	None	Emphysematous change with pleural fibrosis, no evidence of malignancy

Patients 1-4 underwent surgery for diagnostic reasons of ^{18}F-Fluoro-deoxyglucose positron emission tomography (FDG-PET) positive parenchymal lung lesions. In all cases a firm clinical diagnosis of TB was made. All 5 patients improved on appropriate TB therapy. All patients tested were HIV negative and there were no cases of multidrug resistant TB. Patients C1-2 underwent resection of malignant/ non-malignant nodules that were surrounded by relatively normal lung.
